# OLDER ADULTS WITH SCHIZOPHRENIA AND INFLAMMATORY MARKERS OF AGING

**DOI:** 10.1093/geroni/igae098.1077

**Published:** 2024-12-31

**Authors:** Heather Leutwyler, M K Kirsten Chui, Kevin Schneider, Sara LaHue, David Furman, John Newman

**Affiliations:** University of California San Francisco, Pacifica, California, United States; Buck Institute for Research on Aging, Novato, California, United States; Buck Institute for Research on Aging, Novato, California, United States; University of California San Francisco, San Francisco, California, United States; Buck Institute for Research on Aging, Novato, California, United States; Buck Institute for Research on Aging, Novato, California, United States

## Abstract

Schizophrenia is associated with accelerated aging, evidenced by earlier onset of aging-related diseases such as hypertension and geriatric syndromes. Inflammaging is a state of dysfunctional systemic chronic inflammation that accelerates a negative feedback loop with other biological aging mechanisms and increases risk for aging-related chronic diseases. Inflammaging is driven in part by discrete cellular and molecular processes such as the accumulation of senescent cells and their resulting pro-inflammatory senescence-associated secretory phenotype (SASP). We report the results of a cross-sectional analysis from older adults with schizophrenia (OAS) to describe the relationships between health and inflammaging biomarkers. We analyzed plasma from 24 OAS (mean age 59.5). Neurocognition was assessed with the MATRICS Consensus Cognitive Battery and schizophrenia symptoms with the Positive and Negative Syndrome Scale. Physical health was assessed via the physical function subscore of SF-12. We obtained quantitative measurements of 112 cytokines and other factors using the Millipore Milliplex platform (Eve Technologies). We also derived from these data composite measurements of SASP via lists published by Diniz et al. (11 components), SenMayo (35 components) and the Translational Geroscience Network (36 components). Specific inflammatory cytokines (eotaxin, IL-1α, IL-1β, and IFNα) were associated with worse cognition and physical health, while no significant associations were observed with SASP composite scores. These results suggest that specific pathways of inflammaging, which may be driven by biological mechanisms other than cellular senescence, associate with important clinical outcomes in OAS. Larger studies could better characterize aging mechanisms that contribute to inflammaging in the accelerated aging state of OAS.

